# Development and Optimization of Novel Emulgel Loaded with Andrographolide-Rich Extract and Sesame Oil Using Quality by Design Approach: In Silico and In Vitro Cytotoxic Evaluation against A431 Cells

**DOI:** 10.3390/gels9070507

**Published:** 2023-06-21

**Authors:** N. V. L. Sirisha Mulukuri, Moumita Dhara, Dheeraj Gupta, Kusum Devi, Pankaj Kumar

**Affiliations:** 1 Department of Pharmaceutical Chemistry, NGSM Institute of Pharmaceutical Sciences (NGSMIPS), Nitte (Deemed to be University), Mangalore 575018, India; sirisha.mulukuri@nitte.edu.in (N.V.L.S.M.); dj8207@gmail.com (D.G.); 2 Nitte College of Pharmaceutical Sciences, Bangalore 560064, India; moumitadhara1718@gmail.com (M.D.); drvkusum.devi@nitte.edu.in (K.D.)

**Keywords:** *Andrographis paniculata*, anticancer, emulgels, non-melanoma, topical

## Abstract

An epidermoid carcinoma is a form of non-melanoma skin cancer that originates from the outer layer of the skin’s squamous cells. Previous studies have shown that andrographis extract and andrographolide inhibit the growth and proliferation of epidermoid carcinoma cells while also inducing cell cycle arrest and apoptosis. The objective of this study was to improve the anticancer efficacy of the andrographolide-rich extract by delivering it in the form of nanoemulgel. During the formulation of emulgels, sonication, and homogenization were employed, and a 2^2^-factorial design was used to optimize the formulations through the quality by design (QbD) approach. The optimized formulation (AEE8) was subjected to preliminary evaluations along with particle size, drug release, and scanning electron microscopy (SEM) studies. The potential of the optimized emulgel against A431 cell lines was also investigated using MTT assay followed by flow cytometric analysis. The SEM results reveal that the optimized emulgel had a well-defined spherical shape, with a droplet size of 226 ± 1.8 nm, a negative surface charge of −30.1 ± 1.6 mV, and a PDI of 0.157. The cellular data indicate that AEE8 reduced the viability of the A431 cells with an IC_50_ of 16.56 μg/mL, as determined by MTT assay when compared to cells treated with the extract alone. Furthermore, the flow cytometric analysis of the optimized emulgel formulation demonstrated a marked G2/M phase arrest. This finding further supports the effectiveness of the gel in disrupting the cell cycle at the critical G2 and M phases, which are pivotal for cell division and proliferation. This disruption in cell cycle progression can impede the growth and spread of cancer cells, making the gel a promising candidate for anti-skin-cancer therapy. The safety of emulgels (AEE8) was validated through rigorous biocompatibility testing conducted on HDF (human dermal fibroblast) cell lines, ensuring their suitability for use. Considering the potential of the nanoemulgel, particularly AEE8, as demonstrated by its favorable properties and its ability to disrupt the cell cycle, it holds great promise as an innovative approach to treating skin cancer.

## 1. Introduction

Skin cancer arises because of the abnormal growth of skin cells caused by genetic mutations or exposure to UV radiation. It is the 17th most common cancer worldwide. Based on its impairments, skin cancer has been broadly classified into melanoma and non-melanoma, which is commonly seen in people of Caucasian descent. Epidermoid carcinoma is a type of non-melanoma skin cancer that arises from the squamous cells that make up the outer layer of the skin [1]. The incidence of non-melanoma and melanoma skin cancers has risen in recent decades. The World Health Organization has reported that there are 2 to 3 million new cases of non-melanoma skin cancer and 132,000 new cases of melanoma worldwide each year. A431 is a human epidermoid carcinoma cell line that has been extensively used to study the effects of different compounds on skin cancer cells. Studies on A431 cells have helped shed light on the molecular mechanisms underlying the development and progression of epidermoid carcinoma and other types of skin cancer. 

Herbs and supplements are widely used by cancer patients and survivors to reduce their symptoms and enhance their quality of life [2]. The most popular complementary and integrative medicinal (CIM) treatment for cancer patients in the United States is herbal medicine [3]. Widespread self-prescription of herbal supplements during or after cancer therapies is reported by nearly 60% of patients. The desire for safe and effective treatments has led to a growing interest in herbal remedies [4].

Herbal formulations, such as *Andrographis paniculata* (AP), have gained popularity because of their medicinal properties and low incidence of side effects, unlike various drugs. AP has been used in traditional Unani and Ayurvedic formulations to treat conditions such as snake bites, insect bites, diabetes, diarrhea, fever, and malaria [5]. Research has shown that AP has a range of therapeutic effects, including immune stimulation [6], antidiarrheal [7], antihyperglycemic [8], antihepatitis [9], anti-inflammatory [10], anti-HIV [11], and anticancer activity [12], Metabolites of AP have also been found to be effective against skin cancer [13]. The plant’s high terpene content has been explored for its plethora of therapeutic functions [14], specifically, the terpenoid, andrographolide, reported against skin cancer. 

Various studies of *andrographis* extract and andrographolide on A431 cells have found inhibition of the growth and proliferation of epidermoid carcinoma cells and induction of cell cycle arrest and apoptosis [15]. However, there has been limited research on its formulation, particularly for topical use in cancer treatment. To address this, a novel approach was taken to formulate the extract into emulgels, which have good permeation and drug-loading capacity [16] for the effective release of active constituents. Emulgels are topical formulations that combine the properties of emulsions and gels, providing a stable and uniform formulation [17], which can improve the stability, bioavailability, and sustained release of active ingredients [18]. Emulgels can be used for various cosmetic and pharmaceutical applications [19].

Sesame oil has been shown to have chemopreventive effects on skin cancer by counteracting the growth of cancerous cells. Sesamol, a compound found in sesame oil, has been identified as an active component in these chemopreventive properties [20]. Additionally, sesame oil has been demonstrated to have a protective effect against the harmful effects of UV rays when applied topically to the skin [21]. Sesame oil is known for its stability and remarkable antioxidant activity [22]. 

QbD, or quality by design, is an experimental approach that evaluates how different variables affect product quality. The QbD methodology allows for the assessment of both the main effects and interactions among variables in a cost-effective and time-efficient manner. Different techniques can be used to implement the QbD approach, and statistical software can be used to analyze the data obtained [23].

The emulgel formulation was optimized using the quality by design (QbD) approach with a factorial design, which allowed for the evaluation of multiple factors on the response variable, such as drug release. Critical process parameters (CPPs) and critical quality attributes (CQAs) that have significant impacts on product quality were identified, and their effects on the formulation were systematically evaluated through experiments [24]. This approach can help optimize the formulation by ensuring its quality and efficacy. 

The current study involved a novel emulgel formulation containing a blend of sesame oil and andrographolide-rich extract along with carbapol 934 as a gelling agent. 

The emulgels optimized by QbD were then evaluated for their anticancer potential against A431 cell lines using the MTT assay, followed by a cell cycle analysis study on A431 cell lines using flow cytometry. This step helped determine the effectiveness of the formulation in inhibiting the growth and proliferation of epidermoid carcinoma cells. The optimized emulgels were evaluated using in vitro biocompatibility studies with HDF cells to determine the safety profile against normal skin cells.

## 2. Results and Discussion

### 2.1. HPLC Analysis

**Figure 1:**
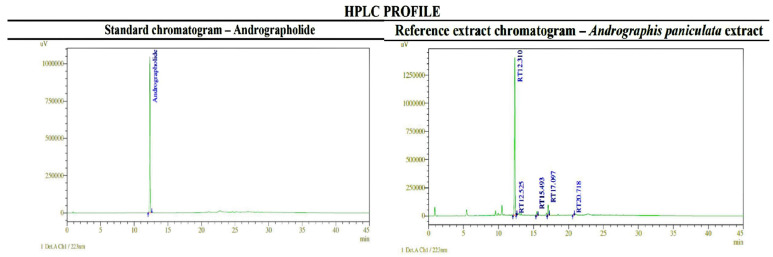
HPLC analyses of *andrographolide and andrographis* extract.

The retention times for the standard andrographolide and the andrographolide-rich extract were, respectively, 12,319 min and 12,310 min for the total run time of 45 min. Comparing the HPLC profiles of the standard andrographolide and the extract (depicted in Figure 1), it was confirmed that the extract contained a significant presence of andrographolide, comprising approximately 63% based on the peak area analysis.

### 2.2. In Silico Studies

**Figure 2:**
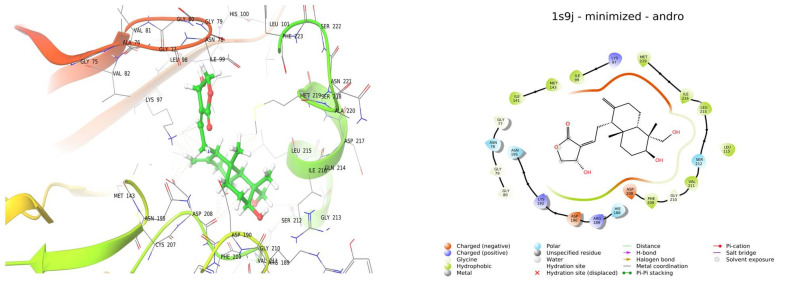
In silico studies of andrographolide.

In silico studies were conducted to evaluate the potential of andrographolide in treating skin cancer. The study used molecular docking to investigate the interaction between andrographolide and specific amino acids in the target protein. Based on the high activity of uridenyl phosphorylase (UrdPh) in melanoma tumor tissue, one potential approach for skin cancer treatment could involve blocking or inhibiting the activity of this enzyme. By blocking UrdPh, it may be possible to disrupt the metabolic processes or signaling pathways that contribute to the growth and progression of melanoma cells, potentially leading to therapeutic benefits in the treatment of skin cancer [25]. Therefore, PDB ID: 1SJ9, corresponding to UrdPh, was selected as the target protein. Surprisingly, andrographolide exhibited the best docking fit, against this target compared to other targets, indicating a strong potential affinity. The obtained docking score of −3.373 reflects the predicted affinity between the ligand (andrographolide) and the target protein (UrdPh). The docking analysis revealed hydrophobic interactions between andrographolide and specific amino acids, including ILE141, MET143, ILE99, LEU115, PHE209, VAL211, LEU215, ILE316, and MET219. Additionally, polar interactions were observed with amino acids ASN78, HIS188, ASN195, and SER212 (Figure 2). The calculated glide energy of andrographolide was determined to be −17,505, indicating a strong predicted binding affinity.

### 2.3. Compatibility Studies

In this study, FTIR observations were conducted to investigate the interactions between the active drug and the excipients used in the formulation. The spectra were obtained for the extract, the main excipients (sesame oil and Carbopol), their physical mixture with the drug, and the test formulations Table 1. The FTIR spectra are given in the Supplementary Materials (Figure S1).

### 2.4. Different Assessment Criteria for Emulgels

#### 2.4.1. Physical Assessment

The formulated emulgels were observed as a greenish viscous substance with a homogenous texture and a glossy appearance. 

#### 2.4.2. pH

Table 2 indicates that the pH values of the emulgels were found to be between 5.8 and 6.2. This suggests that the emulgels may not cause skin irritation.

#### 2.4.3. Viscosity

An increase in the polymer concentration, specifically Carbopol 934, in the formulations resulted in a significant increase in the viscosity, as demonstrated in Table 2 and Table 3. The regression coefficient for the linear equation shows that there was a direct proportionality between the independent variable and direct variables, indicated by a positive value. Conversely, a negative value indicates an inverse relationship between the dependent and independent variables, as shown in Table 2.

The linear equation generated by Design Expert is as follows: viscosity = +0.04020 + 0.0080A + 0.0180B. This equation suggests that an increase in the amount of extract and Carbopol in the emulgel formulations leads to an increase in viscosity, Figure S2.

#### 2.4.4. Spreadability

The results of the spreading coefficients demonstrate that all emulgels had a high degree of Spreadability, as shown in Figure S2. The observed linear equation from the Design Expert is: Spreadability = +28.63 + 0.1250A − 2.13B + 0.3750AB. As the extract and polymer concentration increased, the Spreadability decreased in the formulations, as shown in Table 2 and Table 3.

#### 2.4.5. Extrudability

It was found that all emulgel formulations exhibited good extrudability. The linear equation obtained from Design Expert further supports this finding, as shown in Figure S2. The equation is as follows: extrudability = +77.40 − 0.4000A − 2.90B. This indicates that an increase in the concentrations of both the extract and polymer leads to a decrease in extrudability in the formulations, as shown in Table 2 and Table 3.

### 2.5. In Vitro Diffusion Studies

Figures S2 and S3 illustrate the in vitro diffusion behavior of the formulations. The Carbopol-934-based AEE1-AEE7 formulations exhibited a substantial degree of release, with approximately 80% of the active ingredients released within 10 h. These results suggest that the developed herbal gel formulations have promising in vitro release properties.

### 2.6. Formulation and Evaluation of Optimized andrographolide-Rich Extract-Loaded Emulgel

The software Design Expert was used based on a QbD approach 2^2^ factorial design to develop an optimized emulgel formulation with andrographolide-rich extract. The final composition consisted of 0.08 g extract, 0.5 g carbopol-934, and other excipients. The formulated emulgel underwent various evaluations, including droplet size, polydispersity index (PDI) (as shown in Figure S4), pH, viscosity, spreadability, SEM analysis (depicted in Figure S5), and drug release studies (illustrated in Figure S6). The results for these parameters were statistically comparable, with a droplet size of 226 ± 1.8 nm, PDI of 0.157, pH of 5.78 ± 0.3, a viscosity of 0.384 ± 0.2 poise, and a spreadability of 30.78 g/cm^2^. The drug release study revealed a sustained release of 92.8% of andrographolide over time at pH 5.5 and pH 7.4. Different kinetic models were examined to explain the release pattern, and it was concluded that the optimized emulgel (AEE8) followed zero-order and Hixson−Crowell kinetics, which explains the sustained release observed with the emulgels.

Although all formulated emulgels exhibited good stability, the optimized formulation (AEE8), selected based on preliminary evaluation using the quality by design (QbD) approach, is now eligible for screening for its potential as an anticancer agent. This screening process aims to evaluate its efficacy in inhibiting cell proliferation, inducing apoptosis, or affecting cancer-related pathways. The selection of the optimized formulation for screening signifies its promising characteristics and marks an important step towards assessing its potential as a therapeutic option for cancer treatment.

### 2.7. Anticancer Studies 

#### 2.7.1. Cytotoxic Activity

**Figure 3:**
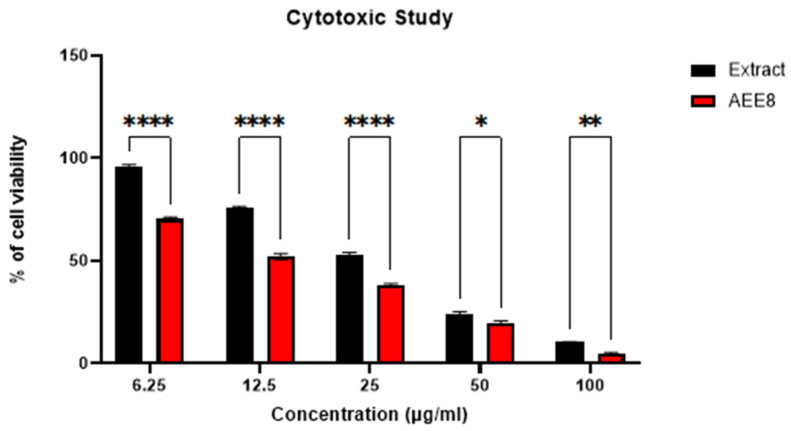
The % cell viability of A431 cells incubated with different concentrations (6.25, 12.5, 25, 50, and 100 μg/mL) of extract and emulgel (AEE8) for 48 h Using a two way ANOVA model, data are presented as the mean ± standard deviations (*n* = 3). **** indicates *p <* 0.001, ** indicates *p <* 0.01, * indicates *p <* 0.05.

**Figure 4:**
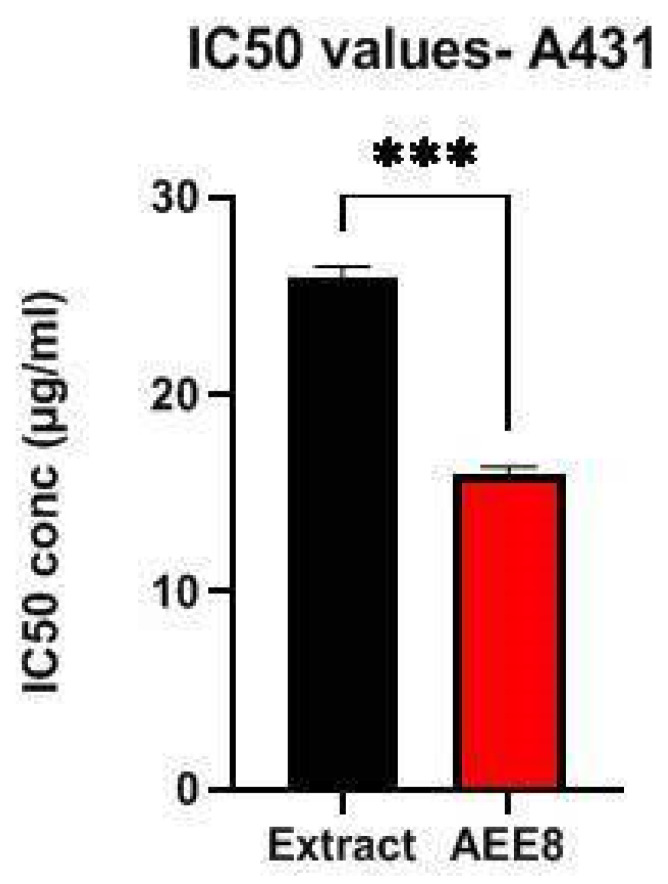
IC_50_ values of extract and AEE8 against A431 cell lines. Using a two way ANOVA model, data are presented as the mean ± standard deviations (*n* = 3). *** indicates *p <* 0.001.

The cytotoxic activity of the optimized formulation, AEE8, was evaluated against A431 cells using the MTT assay. The IC_50_ value for AEE8 was 16.56 µg/mL, which was significantly lower than the IC_50_ value of the extract 26.87 µg/mL. Pure andrographolide with an IC_50_ value of 8.07 µg/mL was used as a standard drug, and the results are presented in Figure 3 and Figure 4.

#### 2.7.2. In Vitro Cell Cycle Assay

A cell cycle assay was performed to conduct a cell cycle analysis using propidium iodide (PI). Based on the significant cell inhibition observed with the andrographolide, extract, and AEE8 emulgel after a 24 h treatment period, the IC_50_ concentration was selected for further evaluation against A431 cell lines. To investigate the effects on the cell cycle a cell cycle study was conducted using flow cytometry. The results obtained from the flow cytometry analysis are presented below Table 4.

**Figure 5:**
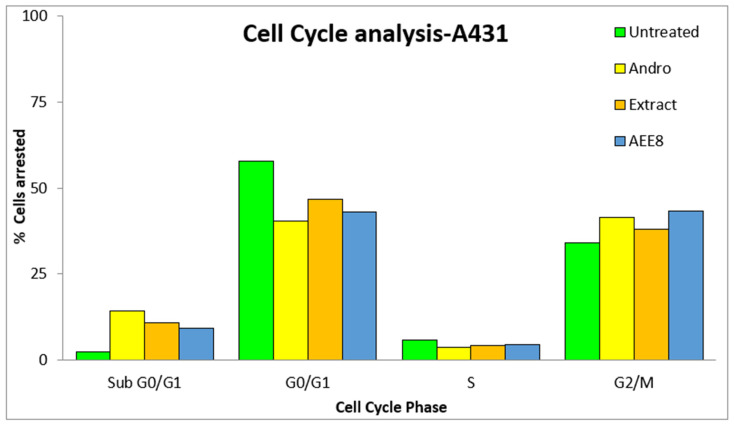
Overlaid bar graph showing the % cells arrested or distributed in the different phases of the A431 cell cycle upon treatment with andrographolide, extract, and gel in comparison.

**Figure 6:**
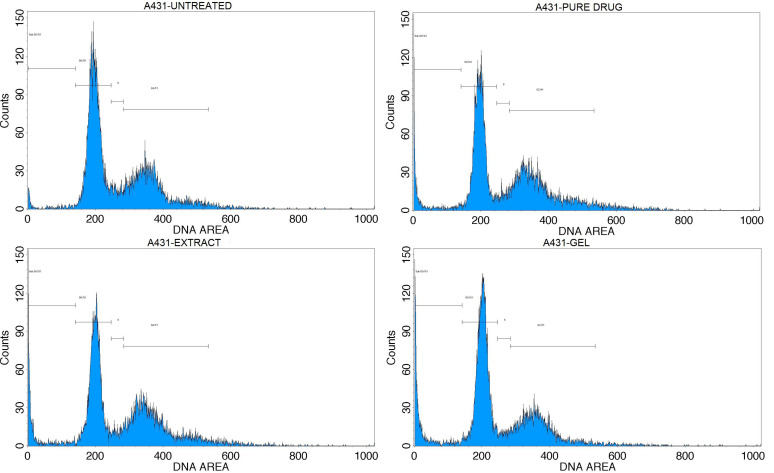
Flow cytometric histograms representing the phases of cell cycle distribution in the A431 cell line—untreated, andrographolide, extract, and AEE8 with IC_50_ values in comparison.

In the sub G0/G1 phase (apoptotic phase), 2.35%, 14.38%, 10.97%, and 9.32% cells were arrested in the untreated, andrographolide, extract, and gel with IC_50_ concentrations, respectively. In the G0/G1 phase (growth Phase), 57.74%, 40.42%, 46.81%, and 42.9% of cells were arrested in the untreated, andrographolide, extract, and AEE8 with IC_50_ concentrations, respectively. In the S phase (synthetic phase), 5.82%, 3.79%, 4.2%, and 4.57% of cells were arrested in untreated, andrographolide, extract, and AEE8 with IC_50_ concentrations, respectively. On the other hand, in the G2/M phase, 34.09%, 41.41%, 38.02%, and 43.21% cells were arrested in the untreated, andrographolide, extract, and AEE8 with IC_50_ concentrations, respectively depicted in Figure 5 and Figure 6. 

### 2.8. In Vitro Biocompatibility Analysis

**Figure 7:**
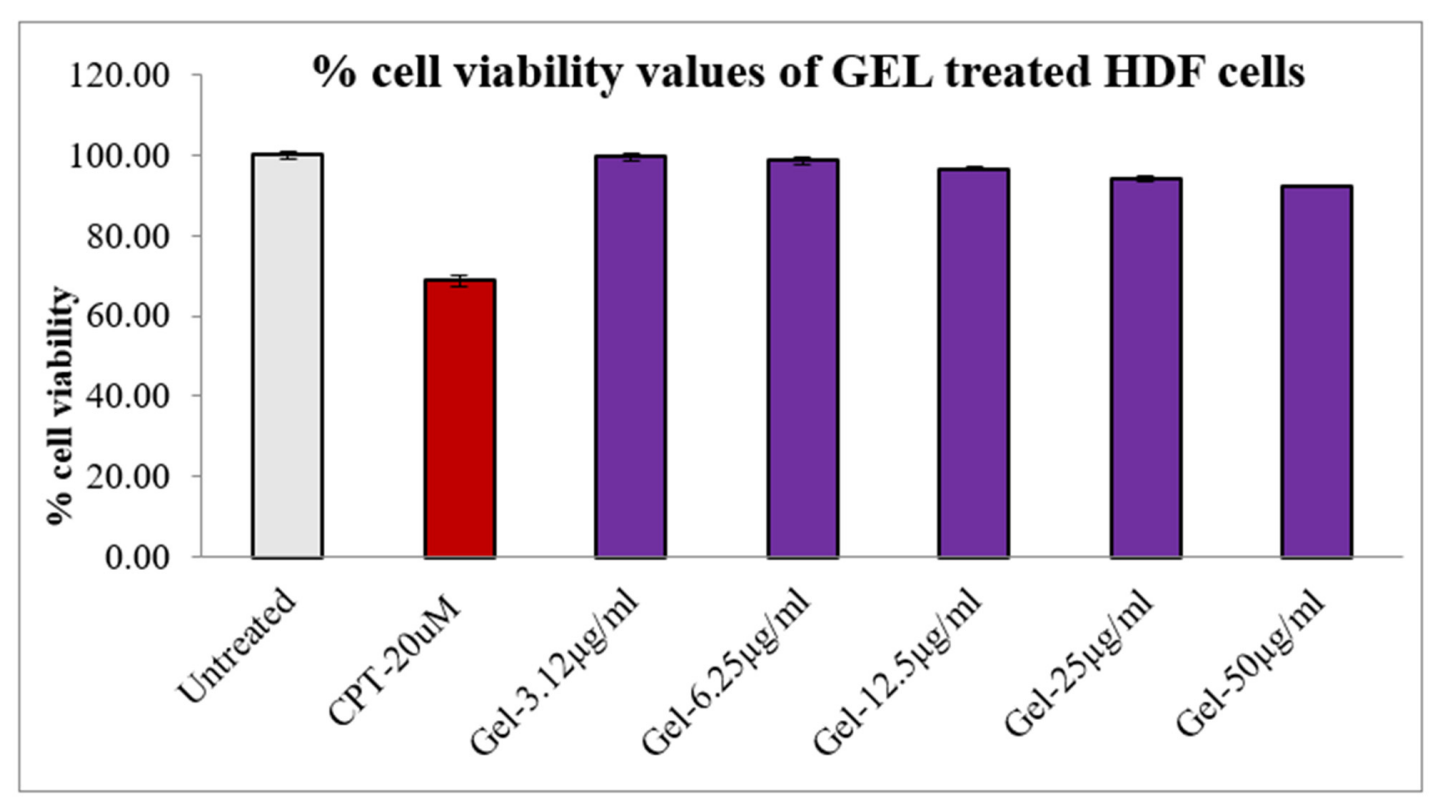
The % cell viability of gel with various concentrations on treated HDF cells after an incubation period of 24 h.

The results of cytotoxicity study performed by MTT assay suggest that given andrographolide, emulgel (AEE8) was nontoxic in nature on dermal fibroblasts (HDFs) with 90.54% and 92.37% cell viability values after the 24 h of incubation, as shown in Table 5 and Figure 7. The safety and biocompatibility of AEE8 is likely attributed to its nanoemulsion-based structure, which allows for better dispersion and interaction with biological components. The nanoscale size and stability of the emulgels likely facilitate efficient cellular uptake and minimize any potential toxic effects Table 5.

### 2.9. Statistical Analysis

MS Excel and SPSS software were used in the current research work for statistical data analysis, where the *p* values were < 0.05.

## 3. Conclusions

Currently, there is no phytotherapy available for the topical treatment of non-melanoma skin cancer. To address this gap, an attempt was made to topically administer andrographis extract using a nanoemulgel formulation, representing a novel method for delivering phytoactive therapy to patients with dermal cancer that may provide substantial benefits. The ethanolic fraction of *Andrographis paniculata* contains terpenoids, primarily andrographolide, which has been shown to exhibit anticancer activity in the current investigation. The optimized emulgel formulation, AEE8, containing 0.08 percent extract and 0.5 percent Carbopol-934, has demonstrated acceptable effects against A-431 cancer cells. The formulated nanoemulgel AEE8 has demonstrated greater cytotoxic properties against A431 cell lines compared to the Extract. Additionally, it has shown a significant ability to induce cell cycle arrest, similar to andrographolide. The evaluation of safety and biocompatibility is crucial when considering the potential clinical applications of any new material or formulation. The results obtained from the study indicate that the prepared nanoemulgels, specifically AEE8, demonstrate a high level of safety and biocompatibility. Therefore, the emulgel formulation concept proves to be a valuable approach for enhancing the effectiveness of andrographis extract in topical applications. AEE8 has the potential to be developed as a therapeutic agent for the treatment of skin cancer. The successful development of a topical treatment option for non-melanoma skin cancer utilizing andrographis extract represents a promising advancement in the field of phytotherapy.

## 4. Materials and Methods

### 4.1. Materials 

The leaves of *Andrographis paniculata* were gathered from the gardens of Tirupathi, authenticated with FRLHT (Bangalore), voucher no. 123919. Human skin adenocarcinoma cell lines (A431) and HDF (human dermal fibroblast cell line) (ATCC, Manassas, VA, USA) were procured from the National Centre for Cell Lines (Pune, India). Carbopol-934 and glycerin were acquired from Loba Chemicals (Mumbai, India.), and propylene glycol, propylparaben, and methylparaben were obtained from Vasa Chemicals (Bangalore, India). Triethanolamine was received from Merck Chemicals (Mumbai, India), and EDTA and DMSO were acquired from Merck (Berlin, Germany).

### 4.2. Preparation of Andrographis Paniculata Extract

The fresh leaves were collected and dried at 40 °C followed by pulverization. The resulting powder was subjected to the defatting process using petroleum ether. Following the defatting process, the plant material was subjected to Soxhlet extraction for 24 h using ethanol as the solvent. The obtained extract was placed for the Rota evaporation process, followed by labeling, and stored in the refrigerator followed by the phytochemical screening [25].

### 4.3. HPLC Analysis: Determination of Andrographolide in the Extract

The samples were analyzed with an auto-sampler HPLC (Merck, Berlin, Germany) LichroCART, Lichrospher, and C18-C18-5µ column. The mobile phase used was buffer:0.01 N potassium dihydrogen phosphate in water +0.5 mL orthophosphoric acid and acetonitrile, with an injection volume of 1.5 mL/min, with UV detection at 223 nm. The filtration of the samples was conducted using a 0.45 μm syringe filter, where standard 0.5 mg/mL andrographolide in methanol and 5 mg/mL sample extract in methanol were injected.

### 4.4. In Silico Studies

The process of molecular docking for the bioactive compound was performed using the Schrodinger docking software with automated capabilities. The ligand was imported from the PubChem portal, and the ligprep file was generated using the Maestro tool version 4.2 (Schrödinger, New York, NY, USA); PDB ID: 1SJ9 was retrieved from the rcsb.org portal, and the missing loops and missing amino acid residue were add and minimized. Further glide grids were generated, and finally the ligand was docked [26].

### 4.5. Compatibility Studies

To investigate the chemical interactions between the andrographis extract and the excipients in the emulgel formulations, Fourier-transform infrared spectroscopy (FTIR) was conducted. The analysis was carried out using an FTIR spectrophotometer (Bruker-(Alpha), Ettlingen, Germany) via the KBr pellet technique in an inert atmospheric condition covering a wave range of 4000–400 cm^−1^. The samples analyzed comprised the andrographis extract, drug excipients, such as sesame oil and Carbopol, their physical mixture, and the emulgel formulation [27]. 

### 4.6. Preparation of Emulgels

The modified method of the process was employed for the formulation of emulgels [28]. The selection of the oil is an important parameter for the formation of a stable emulsion. Sesame oil was selected, based on the optimum solubility of the extract, which was the unique aspect of this formulation, instead of using synthetic oils, to reduce potential side effects. Sesame oil is known to be an effective topical protective agent [29] with good penetration capacity along with an anticancerous effect, making it a synergistic addition to the emulgel preparation along with triethanolamine for the release of bioactive agents. The gel base was prepared by combining the required amount of Carbopol-934 (0.5–1.5%) with water and mixing it until a homogenous gel was obtained. Triethanolamine was added to regulate the pH to a range of 5.85–6.35. To prepare the oil phase, span 80 (0.5%) was dissolved in the Sesame oil (3.75%) and the extract was added (0.04–0.12%). For the aqueous phase, Tween 80 (0.25%), propylene glycol (2.5%), glycerin (1.25%), and methylparaben (0.01%) were dispersed in the appropriate amounts of water. The oil and aqueous phases were heated to a temperature of 40–50 °C, and the oily phase was added dropwise to the aqueous phase by stirring until the mixture reached room temperature, ensuring a uniform emulsion. To prepare the emulgel, the gel and emulsion were blended in equal parts using a homogenizer with suitable speed settings. Fixed concentrations for all ingredients were used. The concentrations of extract and Carbopol-934 varied as per the design obtained using Design Expert software (Table 6). 

### 4.7. Formulation Design Using Design Expert (Central Composite Design)

The 2^2^ factorial approach was selected to optimize the formulation factors with the evaluation parameters statistically. A 2-level factorial design with 3 center points was constructed to explore the response surfaces using Design Expert software (Version 13). Extract, A 0.04 (−1, low level) and 0.12 (+1, high level), Carbopol, B 0.5 (−1, low level), and 2.5 (+1, high level) were taken. Factors and responses were provided in Table 7. 

### 4.8. Evaluation Parameters

#### 4.8.1. Physical Examination

The emulgel formulation underwent periodic evaluation to determine quality control parameters, such as color, odor, texture, consistency, stickiness, and phase separation.

#### 4.8.2. pH

Measuring the pH of the emulgel formulation is a crucial step in determining its quality and efficacy. The pH measurement provides important information regarding the acidity, neutrality, or alkalinity of the formulation, which can impact its effectiveness and stability. To determine the pH of the emulgel formulations, a digital pH meter (RPB1000, India) was utilized after calibration at room temperature. A 1% solution of the emulgel formulation was placed into a glass beaker, and the pH was measured by inserting the electrode of the pH meter into the solution. To ensure accuracy, triplicate readings were taken, and the mean and standard deviations were calculated [30]. 

#### 4.8.3. Viscosity

Measuring viscosity is a critical step in assessing the emulgel formulations, as it can affect their application and efficacy. The Brookfield digital viscometer is widely used for viscosity measurements because of its accuracy and reliability. The use of Spindle 6 and a rotation speed of ten revolutions per minute is a standard method for determining viscosity. Additionally, using a container with a wide aperture allows for the proper insertion of the viscometer spindle into the emulgel sample, which can improve the accuracy of the measurements [31]. 

#### 4.8.4. Spreadability

To measure the spreadability of an agent, researchers employed a pulley-equipped wooden block, a pair of mirror-image glass slides, and some standard weights. Five minutes of pressure from a weight of 1000 g were used to evenly distribute the gel throughout the glass slides. The gel formulation was applied to the glass slide at a weight of 1 g, and the slide was then covered with a second glass slide. A lower glass slide was secured to a bracket, while an upper glass slide was suspended freely from a pulley loaded with 20 g weights. The spreadability by timing how long it took the top glass slide to glide downwards under load from a height of 7.50 inches was measured [32].

S = M × L/T

(where S, M, L, and T are spreadability, the weight of the pan, distance moved by the glass slide, and time required to separate glass slides from one another, respectively).

#### 4.8.5. Extrudability

The extrudability of emulgels refers to the ability of the product to be extruded from its packaging. It is determined by measuring the force required to extrude a 0.5 cm ribbon of the emulgel from a detachable lacquered aluminum tube within a ten-second time frame. This test is conducted multiple times to obtain accurate readings, and an average is calculated to determine the emulgel’s extrudability [33].

Extrudability = Weight subjected for the extrusion of the gel (g)/Area (cm)

#### 4.8.6. In Vitro Diffusion Studies

Diffusion studies of emulgels typically involve investigating the rate and extent of drug release from emulgels, which are topical formulations consisting of both water and oil phases stabilized by an emulsifier. A drug release study was conducted using a Franz diffusion cell. The experimental emulgels were applied onto a dialysis membrane in a predetermined quantity and placed onto the Franz diffusion cell. To simulate medicament release, 25 mL of buffer (pH 7.4) was injected into the bottom compartment of the cell. The set-up was placed on a magnetic stirrer for continuous stirring at 37 °C. Blank readings were taken for comparison, and at regular intervals of 30 min, 1 mL of release media was withdrawn and replaced simultaneously with fresh medium to maintain sink conditions. The experiment was conducted for 8 h, and the collected samples were adequately diluted to measure the absorbance using UV-spectrophotometric analysis at 223 nm (Shimadzu 1900, Mumbai, India) by a standard plot followed by computation of cumulative percentage drug release values [28].

### 4.9. Anticancer Studies

#### 4.9.1. Cytotoxic Activity

To evaluate the potential cytotoxic effects of various concentrations (6.25, 12.5, 25, 50, and 100 µg/mL) of andrographis extract emulgel formulations on A-431 cell lines, the MTT assay was utilized. A-431 cells (2 × 104) were seeded into each well of a 96-well tissue culture plate containing 100 µL of DMEM and incubated at 37 °C with 5% CO_2_ for one day. After the cells were treated with the emulgel formulations, 5 mg/mL MTT solution in PBS was added (20 µL) to each well and incubated for 4 h at 37 °C. Subsequently, the formazan crystals formed were dissolved in 100 µL of DMSO, and the optical densities were measured at 570 nm using an ELISA reader (Bio-Rad, Hercules, CA, USA). The experiment was repeated three times, and the average readings were recorded. The specific absorbance was calculated by subtracting the absorbance of the solvent from the overall absorbance to obtain accurate results [34].

#### 4.9.2. In Vitro Cell Cycle Assay

In order to quantify the distribution of cells in different phases of the cell cycle, including sub G1, G1, S, and G2/M, a cell cycle assay was performed. A431 cells were plated onto six-well plates at a density of 2 × 10^5^ cells per well and cultured in DMEM supplemented with 10% FBS. After allowing 24 h for adhesion, the cells were treated with IC_50_ concentrations of andrographolide, extract, and AEE8 for 24 h. Following the treatment, the cells were harvested and fixed. Subsequently, they were rinsed with PBS and stained with propidium iodide and RNase in PBS at room temperature for 30 min. Finally, the samples were analyzed using flow cytometry (BD FACS Calibur, BD Biosciences, San Jose, CA, USA).

## 5. In Vitro Biocompatibility Analysis

The optimized nanoemulgel, AEE8, containing andrographis extract, underwent cell viability testing using HDF (human dermal fibroblast) cell lines. The cells were cultured in DMEM and seeded in 96-well plates at a density of 20,000 cells per well. After 24 h of incubation in a 5% CO_2_ atmosphere at 37 °C, the emulgel with different concentrations diluted in culture media (3.125, 6.25, 12.5, 25, and 50 μg/mL) was added to the wells. Following another 24 h incubation period, the spent media were removed, and the MTT reagent was added at a final concentration of 0.5 mg/mL. After removing the MTT reagent, 100 μL of solubilization solution (DMSO) was added and gently stirred on a gyratory shaker to enhance dissolution. Finally, the absorbance was measured at a wavelength of 570 nm using a spectrophotometer or an ELISA reader.

## Figures and Tables

**Table 1:** IR values of specific functional groups.

Sample	Peak/Band Observed	Wavenumber (cm^−1^)
Carbopol 934	Ketone or aldehyde C=O stretching	1710.25
	C=C stretching	1514
	CH_2_ bending and CH_3_ bending	1452
	C-O-C stretching	1173, 1048
	Alkane C-H stretching	2951
	Alcohol C-O stretching	1242
Extract	Amine C-N stretching	1076
	Alkene C=C stretching	1409
	Aromatics C=C stretching	1593.81
	Alcohol O-H stretching	3312
Sesame oil	Phenolic OH	3661
	Unsaturated CH stretching	3007.42
	Saturated CH stretching	2922
	CH bending	1463

Top of Form.

**Table 2:** Evaluation parameters.

	AEE1 *	AEE2 *	AEE3 *	AEE4 *	AEE5 *	AEE6 *	AEE7 *
**Viscosity (poise)**	0.382 ± 0.5	0.375 ± 0.4	0.389 ± 0.2	0.356 ± 0.4	0.368 ±0.2	0.353 ± 0.3	0.372 ± 0.4
**Extrudability ** **(% *w*/*w*)**	76 ± 4	80.3 ± 3.2	81 ± 3.7	84.03 ± 3	81.2 ± 4	85.6 ± 4	81.2 ± 4.7
**Spreadability (g·cm/s)**	27 ± 2	29 ± 5	29.33 ± 2	30.5 ± 1.2	29.6 ± 4	31 ± 3	29.4 ± 2.9
**Drug content** **(% *w*/*w*)**	79.24 ± 0.6	77.19 ± 0.1	80.78 ± 0.3	81.24 ± 0.6	79.59 ± 0.3	82.68 ± 0.4	80.39 ± 0.1
**pH**	5.96 ± 0.6	6.18 ± 0.4	6.33 ± 0.6	5.85 ± 0.5	6.19 ± 0.4	6.35 ± 0.3	6.20 ± 0.3

* Represents the mean ± S.D of three observations.

**Table 3:** Responses: (a) Carbopol; (b) Extract on responses—viscosity, extrudability, spreadability, and drug release (1 h and 8 h).

Source	Sum of Squares	df	Mean Square	F-Value	*p*-Value	Significance
Response 1: Viscosity						
Model	0.0016	2	0.0008	10.78	0.0427	Significant
A-extract	0.0003	1	0.0003	3.56	0.1558	
B-Carbopol	0.0013	1	0.0013	18	0.024	
Residual	0.0002	3	0.0001			
Lack of Fit	0	1	0	0.16	0.7278	Not Significant
Pure Error	0.0002	2	0.0001			
Response 2: Extrudability						
Model	34.28	2	17.14	1101.9	<0.0001	Significant
A-extract	0.64	1	0.64	41.14	0.0077	
B-Carbopol	33.64	1	33.64	2162.6	<0.0001	
Residual	0.0467	3	0.0156			
Lack of Fit	0.04	1	0.04	12	0.0742	Not Significant
Pure Error	0.0067	2	0.0033			
Response 3: Spreadability						
Model	18.69	3	6.23	66.74	0.0148	Significant
A-Extract	0.0625	1	0.0625	0.6696	0.4992	
B-Carbopol	18.06	1	18.06	193.53	0.0051	
Residual	0.7492	3	0.2497			
Lack of Fit	0.5625	1	0.5625	6.03	0.1335	Not Significant
Pure Error	0.1867	2	0.0933			
Response 4: Drug Release: 1 h						
Model	2.5	2	1.25	1137.5	<0.0001	Significant
A-Extract	0.0156	1	0.0156	14.24	0.0326	
B-Carbopol	2.48	1	2.48	2260.8	<0.0001	
Residual	0.0033	3	0.0011			
Lack of Fit	0.002	1	0.002	3.2	0.2157	Not Significant
Pure Error	0.0013	2	0.0006			
Response 5: Drug Release: 8 h						
Model	53.64	2	26.82	185.32	0.0007	Significant
A-Extract	17.64	1	17.64	121.89	0.0016	
B-Carbopol	36	1	36	248.75	0.0006	
Residual	0.4342	3	0.1447			
Lack of Fit	0.3969	1	0.3969	21.3	0.0439	Significant
Pure Error	0.0373	2	0.0186			

**Table 4:** The % cells arrested in the different phases of the A431 cell cycle.

Cell Cycle Study-A431					
Sl. No	Cell Cycle Stage	Untreated	Andro.	Extract	AEE8
1	Sub G0/G1	2.35	14.38	10.97	9.32
2	G0/G1	57.74	40.42	46.81	42.9
3	S	5.82	3.79	4.2	4.57
4	G2/M	34.09	41.41	38.02	43.21
		100	100	100	100
	Total Events Selected	10,000	10,000	10,000	10,000

**Table 5:** The % cell viability of AEE8 and pure drug (andrographolide) against HDF cells after a treatment period of 24 h.

Culture Condition (Concentration)	% Cell Viability AEE8	% Cell Viability Andrographolide
Untreated	100.00	100.00
CPT-20 uM	68.76	68.76
3.12 µg/mL	99.52	99.57
6.25 µg/mL	98.71	99.19
12.5 µg/mL	96.67	98.17
25 µg/mL	94.19	92.63
50 µg/mL	92.37	90.54

**Table 6:** Development of andrographis topical nanoemulgel (% *w*/*w*).

Ingredient (% *w*/*w*)	Formulation Code						
	AEE1	AEE2	AEE3	AEE4	AEE5	AEE6	AEE7
**Extract**	0.12	0.08	0.04	0.12	0.08	0.04	0.08
**Carbopol**	2.5	1.5	2.5	0.5	1.5	0.5	1.5

**Table 7:** Factors and responses in a central composite design include both actual and coded values.

Formulation Code	Values of Factors		Coded Values of Factors	Responses
	Extract (g) (A)	Carbopol (g) (B)		
**AEE1**	0.12	2.5	(+, +)	1. Viscosity 2. Extrudability 3. Spreadability 4. Drug Release: 1 h 5. Drug Release: 8 h
**AEE2**	0.08	1.5	(0, 0)	
**AEE3**	0.04	2.5	(−, +)	
**AEE4**	0.12	0.5	(+, −)	
**AEE5**	0.08	1.5	(0, 0)	
**AEE6**	0.04	0.5	(−, −)	
**AEE7**	0.08	1.5	(0, 0)	

## Data Availability

The article encompases the data.

## Supplementary Materials

The following supporting information can be downloaded at: https://www.mdpi.com/article/10.3390/gels9070507/s1, Figure S1: Compatibility studies of (A) Carbopol; (B) extract; (C) sesame oil; (D) physical mixture; (E) Emulgel; Figure S2. Response surface plot showing the effect of (a) Carbopol; (b) extract on responses—viscosity, extrudability, spreadability, and drug release (1 hr and 8 hrs); Figure S3. In vitro drug release profile of AEE1–AEE7; Figure S4. Particle size determination; Figure S5. Surface morphology of the optimized emulgel; Figure S6. Drug release studies of optimized formulation.
