# Supplementary material for: Development and Optimization of Novel Emulgel Loaded with Andrographolide-Rich Extract and Sesame Oil Using Quality by Design Approach: In Silico and In Vitro Cytotoxic Evaluation against A431 Cells
Source: Gels. 2023 Jun 21;9(7):507. doi: 10.3390/gels9070507 (PMC10379390; doi:10.3390/gels9070507)
Supplement: Supplementary file 1 [file gels-09-00507-s001.zip › gels-2422323-supplementary.pdf]

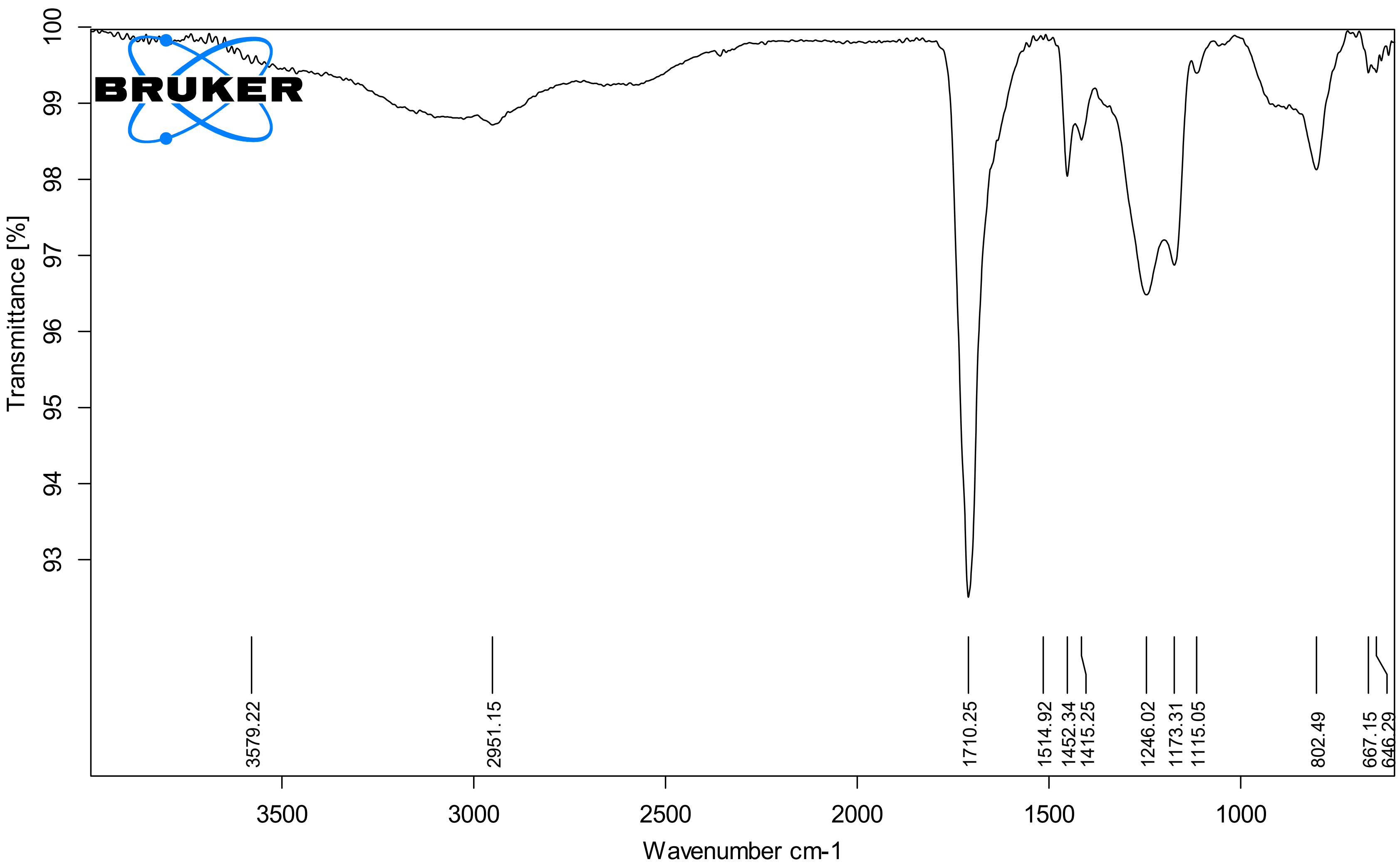

C:\Users\Public\Documents\Bruker\OPUS_8.7.41\MEAS\Carbopol.0	Carbopol	sample	01-03-2023

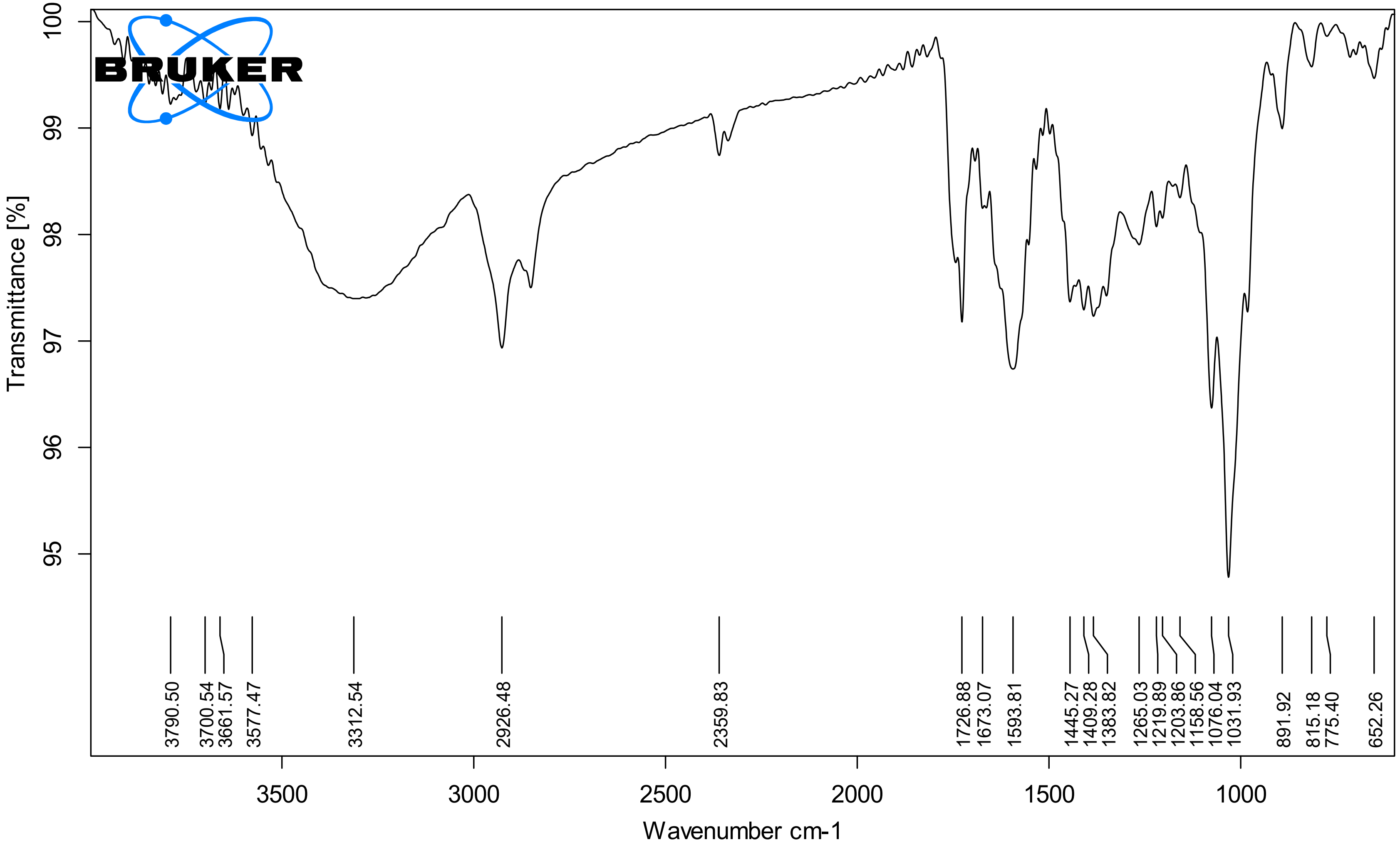

C:\Users\Public\Documents\Bruker\OPUS_8.7.41\MEAS\Plant Extract.0	Plant Extract	sample	01-03-2023

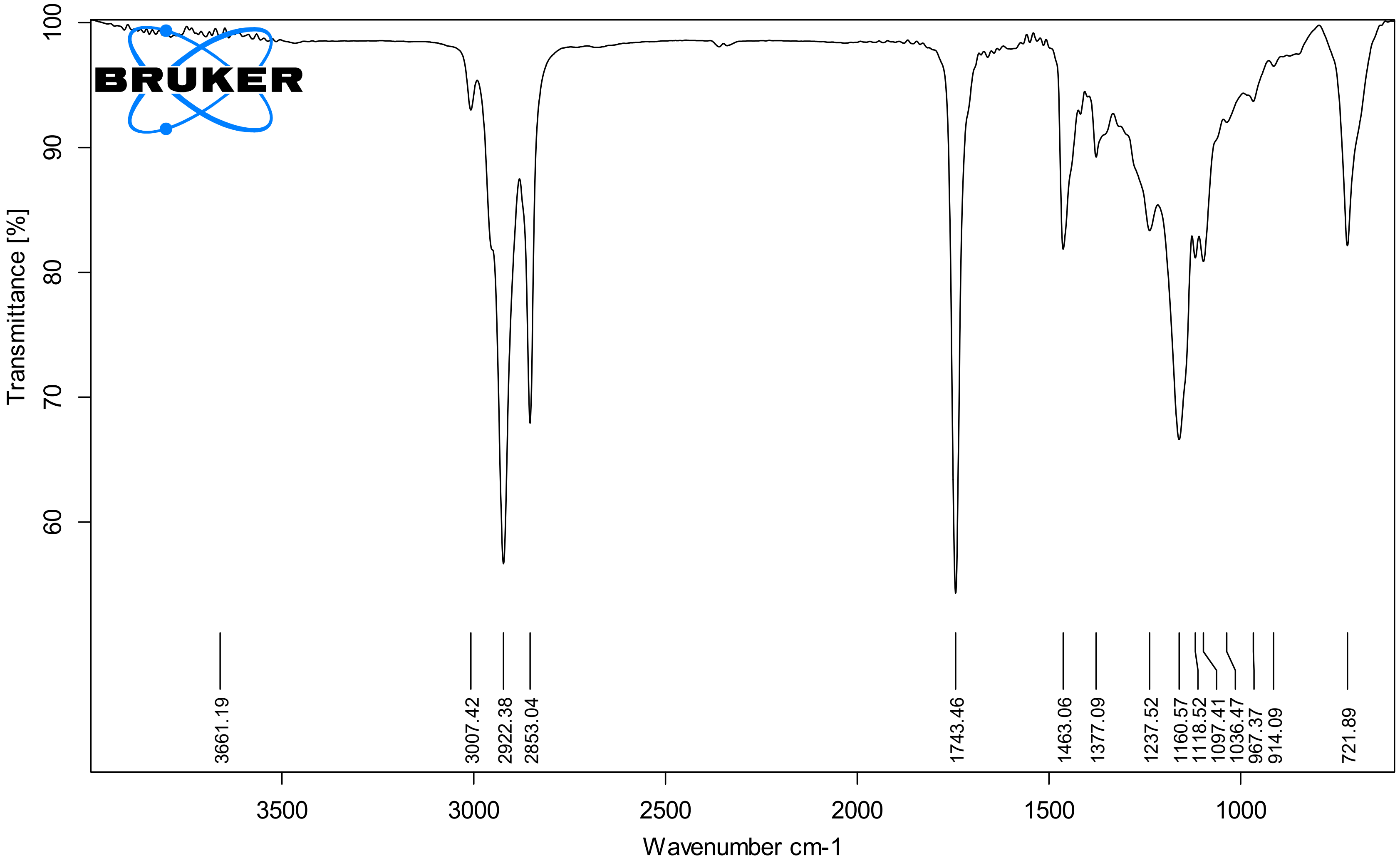

C:\Users\Public\Documents\Bruker\OPUS_8.7.41\MEAS\Til oil.0	Til oil	sample	01-03-2023

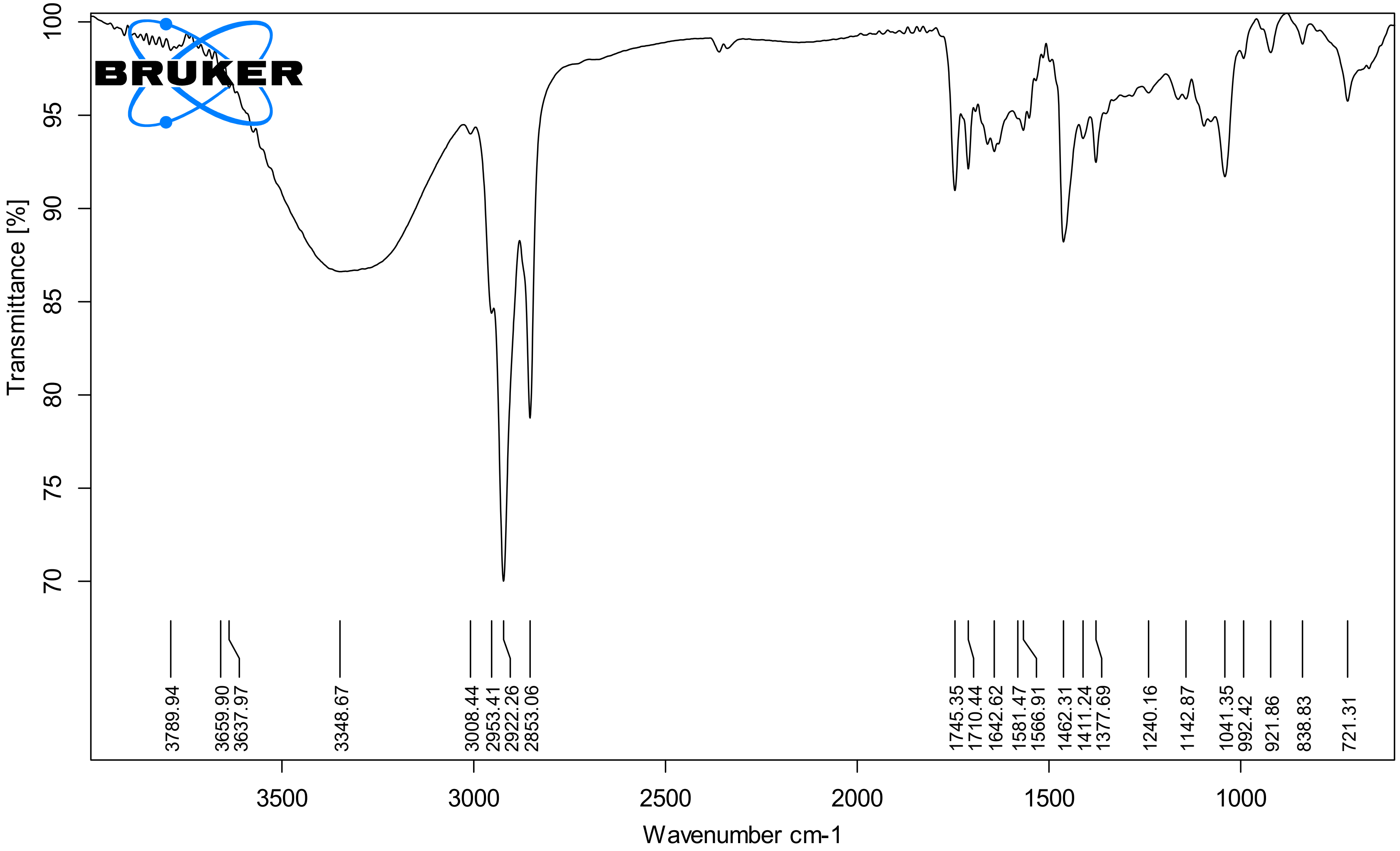

C:\Users\Public\Documents\Bruker\OPUS_8.7.41\MEAS\TE 2.0	TE 2	sample		01-03-2023

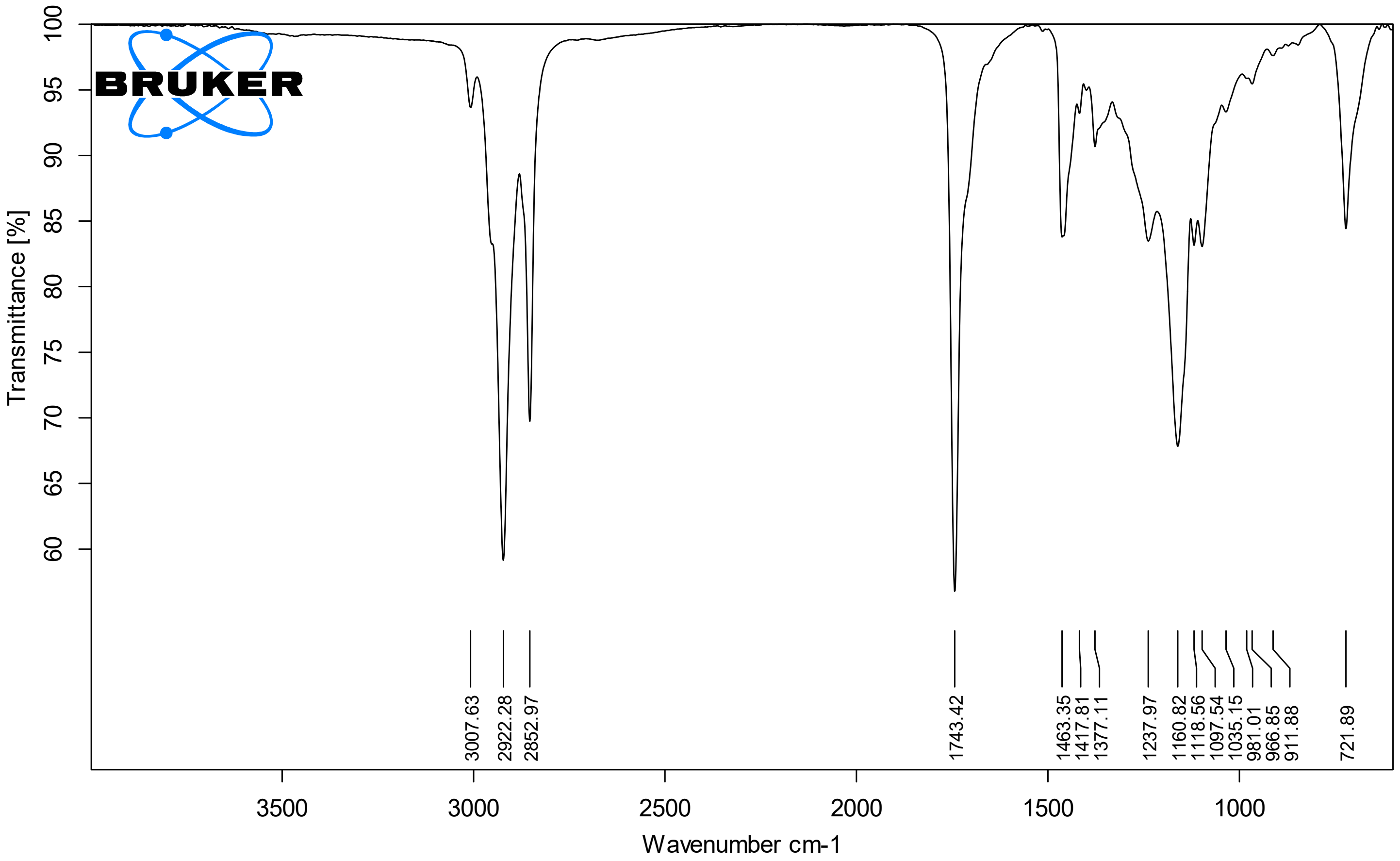

C:\Users\Public\Documents\Bruker\OPUS_8.7.41\MEAS\Physical mixture.1 Physical mixture sample	01-03-2023

Page 1 of 1

Figure S1. Compatibility studies of (A) Carbopol; (B) extract; (C) sesame oil; (D) physical mixture; (E) Emulgel

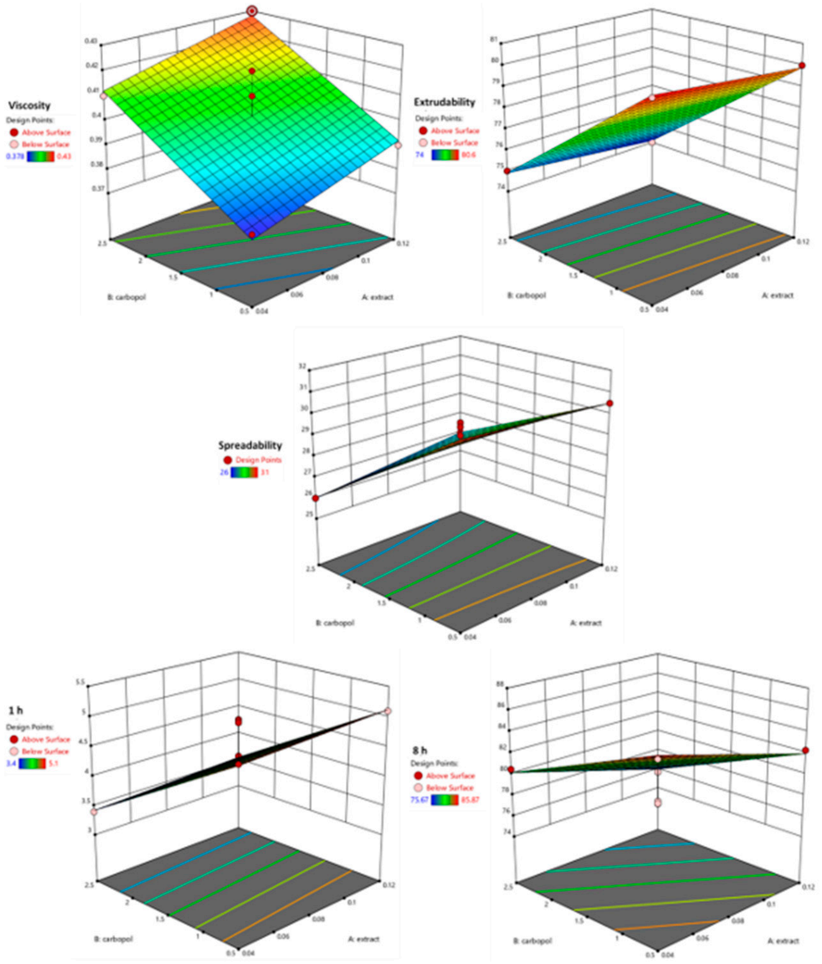

Figure S2. Response surface plot showing the effect of (a) Carbopol; (b) extract on responses—viscosity, extrudability, spreadability, and drug release (1 hr and 8 hrs).

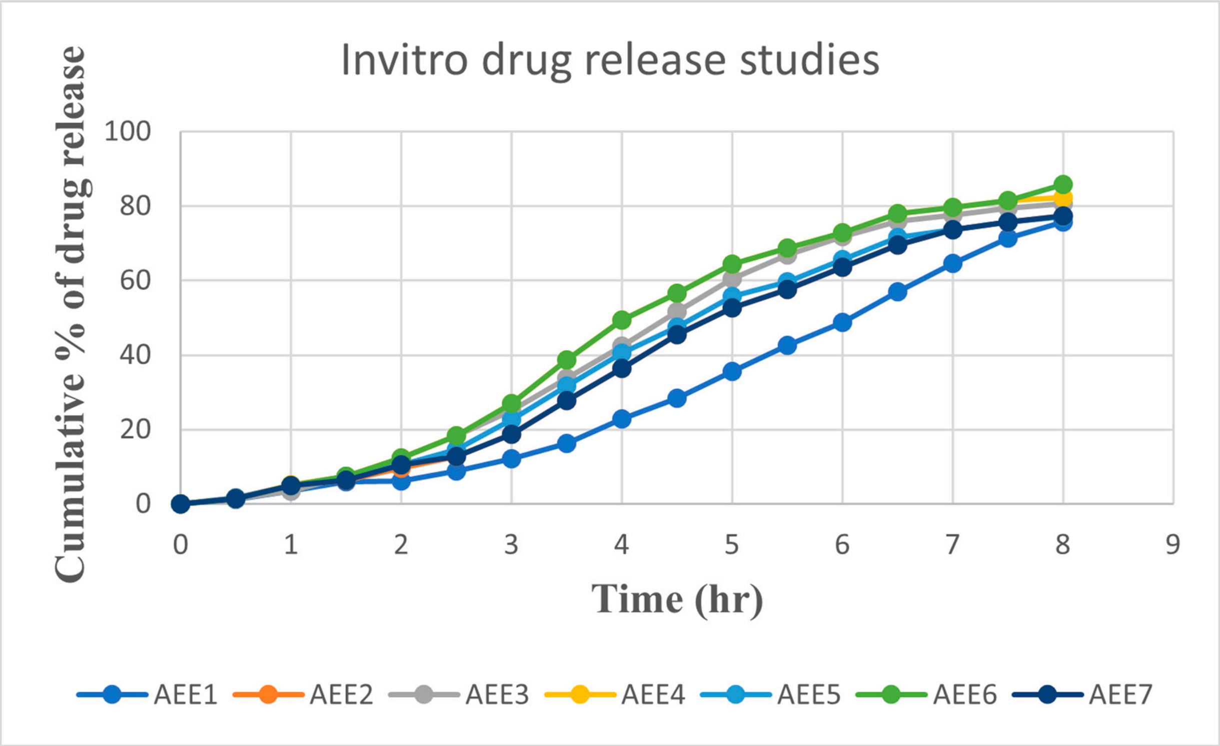

Figure S3. In vitro drug release profile of AEE1–AEE7.

Results

			Size (d.nm):	% Intensity:	St Dev (d.n...
Z-Average (d.nm):	226.0	Peak 1:	254.7	100.0	95.37
PdI:	0.157	Peak 2:	0.000	0.0	0.000
Intercept:	0.903	Peak 3:	0.000	0.0	0.000
Result quality :	Good				

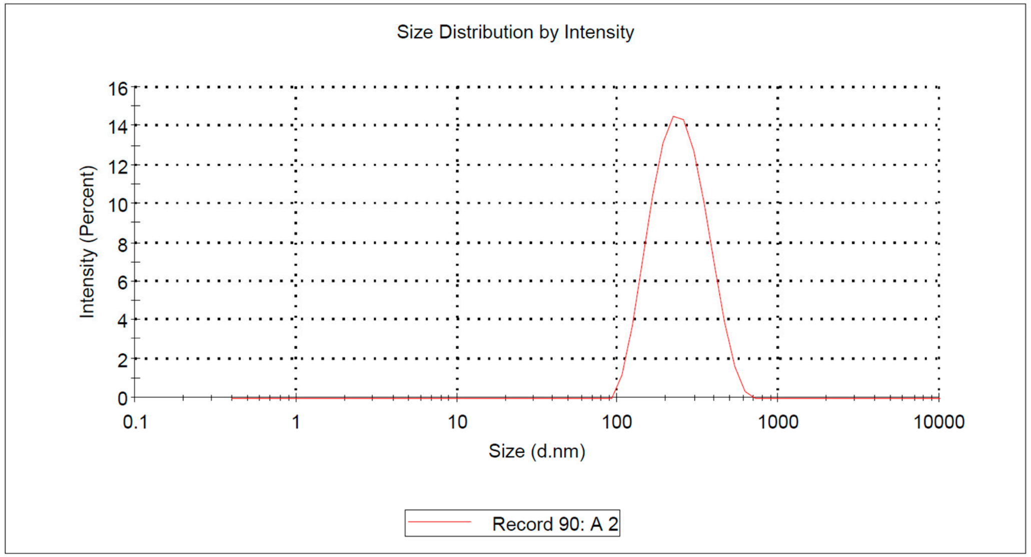

Figure S4. Particle size determination.

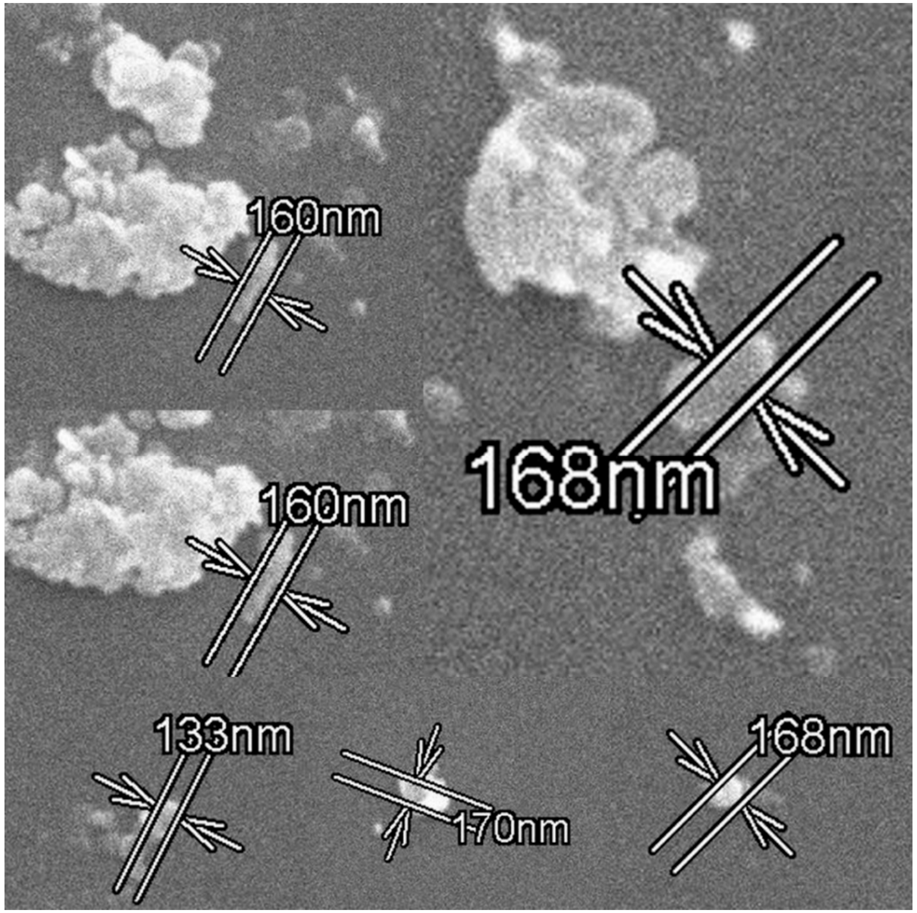

Figure S5. Surface morphology of the optimized emulgel.

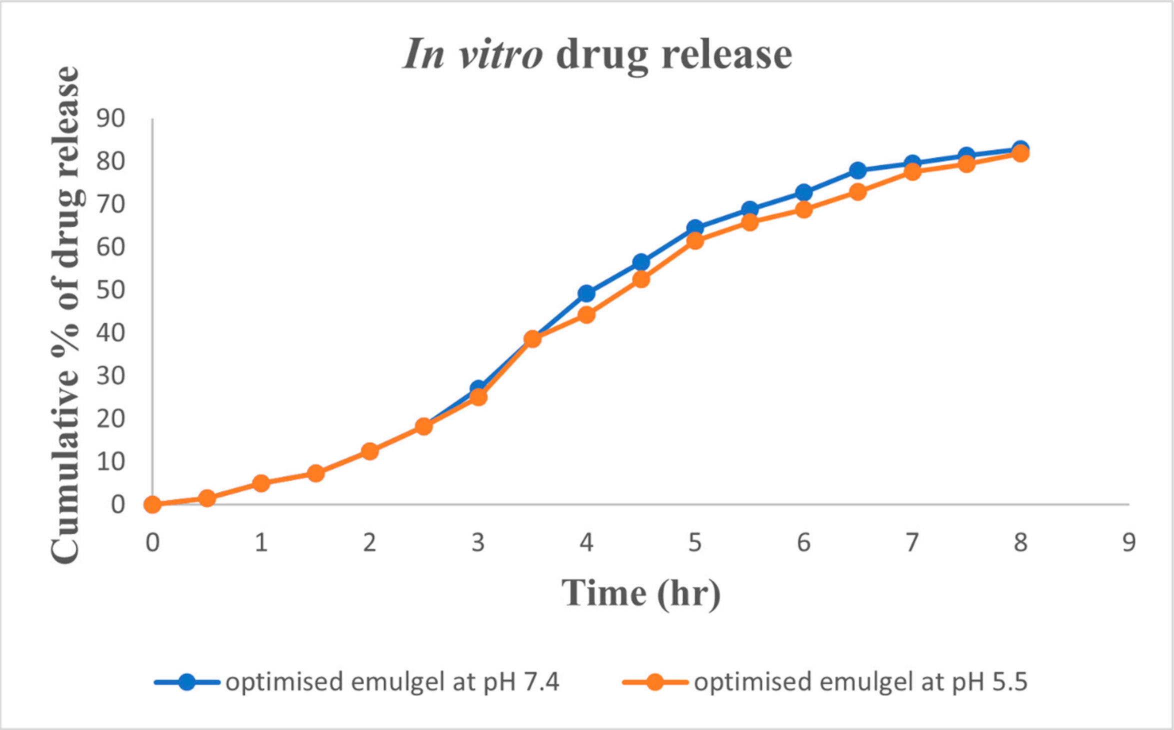

Figure S6. Drug release studies of optimized formulation
